# Nanopore sequencing of microbial communities reveals the potential role of sea lice as a reservoir for fish pathogens

**DOI:** 10.1038/s41598-020-59747-0

**Published:** 2020-02-19

**Authors:** Ana Teresa Gonçalves, Rayen Collipal-Matamal, Valentina Valenzuela-Muñoz, Gustavo Nuñez-Acuña, Diego Valenzuela-Miranda, Cristian Gallardo-Escárate

**Affiliations:** 1 Interdisciplinary Center for Aquaculture Research, University of Concepción, Concepción, Chile; 2 Laboratory of Biotechnology and Aquatic Genomics, Center of Biotechnology, University of Concepción, Concepción, Chile

**Keywords:** Next-generation sequencing, Microbiome

## Abstract

*Caligus rogercresseyi* is a copepod ectoparasite with a high prevalence in salmon farms in Chile, causing severe welfare and economic concerns to the sector. Information on the parasite’s underpinning mechanisms to support its life strategy is recently being investigated. Due to the critical role of microbiota, this study aimed to characterize the microbiota community associated with *C*. *rogercresseyi* from different regions with salmon aquaculture in Chile. Using third-generation sequencing with Nanopore technology (MinION) the full 16S rRNA gene from sea lice obtained from 8 areas distributed over the three main aquaculture regions were sequenced. Microbiota of the parasite is mainly comprised of members of phyla Proteobacteria and Bacteroidetes, and a core microbiota community with 147 taxonomical features was identified, and it was present in sea lice from the three regions. This community accounted for 19% of total identified taxa but more than 70% of the total taxonomical abundance, indicating a strong presence in the parasite. Several taxa with bioactive compound secretory capacity were identified, such as members of genus *Pseudoalteromonas* and *Dokdonia*, suggesting a possible role of the lice microbiota during the host infestation processes. Furthermore, the microbiota community was differentially associated with the salmon production, where several potential pathogens such as *Vibrio*, *Tenacibaculum*, and *Aeromonas* in Los Lagos, Aysén, and Magallanes region were identified. Notably, the Chilean salmon industry was initially established in the Los Lagos region but it’s currently moving to the south, where different oceanographic conditions coexist with lice populations. The results originated by this study will serve as foundation to investigate putative role of sea lice as vectors for fish pathogens and also as reservoirs for antibiotic-resistant genes.

## Introduction

The copepod *Caligus rogercresseyi*, a caligid ectoparasite that is commonly found in southern Chilean salmon farms, is one of most impacting sanitary issues of the industry^1,2^. *C*. *rogercresseyi* infestation has a profound effect on fish, producing irritation, loss of scales and skin wounds that may lead to secondary infections, producing strong physiological^3,4^ and molecular responses^2,5^ in the host. These contribute to a decrease in fish health causing an estimated production cost of 1.4USD per kilogram of produced salmon^6^. Despite the different types of available treatments, bath and oral application of delousing drugs are still preferred^7^. However, reduced sensitivity compromising treatment efficiency for organophosphates and pyrethroids have been recently reported in Chile^8^. The usage of advanced molecular biology tools such as high-throughput sequencing allowed a substantial increase of information regarding the parasite’s invasive and defensive strategies within the parasite-host interface, and during exposure to delousing drugs over the past years (reviewed by Gallardo-Escárate, *et al*.^2^). Despite that, to date there is no available information regarding *C*. *rogercresseyi* associated microbiota, however, due to the key role played by these microorganisms in the host’s well physiological functioning including homeostatic regulation, nutritional, metabolic and defensive performance^9,10^, further research is necessary.

In parasites, the associated microbiota has also been reported as a potential reservoir for secondary pathogens^11^. Known fish pathogens have been found in other caligid parasitic copepods such as *Vibrio* sp. found in *Caligus lalandei*^12^, and there are reports referring to the Northern Hemisphere sea louse, *Lepeophtheirus salmonis*, as a potential vector for *Aeromonas salmonicida*^13,14^. In copepods, microbial communities are most abundant close to the mouth and anus, as well as in digestive tract and eggs sacs^15^, and interactions with both transient and permanent epi or endobiotic associated microbiota seem to promote these organisms’ health^16^. Marine copepods have host-specific microbial associations, symbiosis, or even a central natural core microbiome, that has been reported as more dependent of environment rather than diet^16^. In the case of *C*. *rogercresseyi*, information on these associations of core microbiome might bring light to the parasite life strategy and adaptation capacity and provide insights to develop adequate monitoring and control measures.

Evaluation of complex microbial communities has been performed using culture-dependent techniques, but the reduced proportion of bacteria that can be cultured, as well as the inability to reproduce complex microbial environments *in vitro* are limitations that were overcome by deep sequencing techniques. Presently, the most used method is 16S rRNA gene amplicon sequencing but also shotgun whole-genome metagenomics, the latter being more expensive due to requirements of higher sequencing depth^17^. Dalvin, *et al*.^18^ used shotgun metagenomics and reported that the majority of microorganisms associated with *L*. *salmonis* from the Atlantic and the Pacific Ocean belonged to the phyla Bacteriodetes and Proteobacteria, with a reduced incidence of known fish pathogens. On the other hand, Llewellyn, *et al*.^19^ applied 16S amplicon sequencing on the same species, and reported a high incidence of *Vibrio* sp. Although these are the only reports on sea lice microbiota to date, Moisander, *et al*.^16^ reported in copepods a high abundance of organisms from Proteobacteria phylum, as well as Planctomycetes from PVC superphylum also using 16S amplicon sequencing.

The 16S rRNA gene has nine hypervariable regions with different characteristics, and the V3 and V4 regions have been the most sequenced for taxonomy assignation of the microorganisms associated with aquatic organisms^20^, mostly performed on Illumina platforms. This sequencing strategy is based on sequencing by synthesis, and despite being frequently used, it is limited to a specific region of the 16S rRNA gene, reducing the estimation capacity of richness^21^. In this sense, the third-generation sequencing introduced a new technology based on single-molecule analysis, and sequencers have, therefore, the capacity to sequence full molecules or large DNA fragments^22^. In particular, the nanopore technology with the MinION^TM^ system (Oxford Nanopore Technologies) presents an affordable and portable sequencing strategy that has great potential to evaluate microbiota communities. This technology allows the sequencing of the full-16S rRNA gene, increasing the capacity for taxonomical assignation, and it can be performed in real-time with reduced equipment, increasing its potential as a field tool for remote locations^23^. However, despite these advantages, when comparing to short reads sequencing such as Illumina, the error rate is higher^22^, problem that has been progressively mitigated with technological advances and better bioinformatic tools^24,25^. Regarding the study of microbiota, several studies have been dedicated to compare taxonomical analysis derived by this technology with Illumina sequencing, and have shown that error rate has been compensated with larger fragments, providing in some cases higher taxonomical resolution^26,27^. Although the error rate remains the main limitation to the expansion of this sequencing methodology, it has been successfully applied in several species and tissues, identifying the microbiota present in complex samples from terrestrial^28,29^ and aquatic environment^23,30^. Notably, nanopore sequencing has been applied to understand the interactions of human diseases^31^, environmental samples obtained from glacier regions^23^, ocean water column^32^ and to identify the bacterial community associated with harmful microalgae^30^, and these studies demonstrate the potential and applicability of nanopore sequencing for microorganism detection in all environments.

This study aimed to characterize the microbiota community associated with the salmon’s ectoparasite *Caligus rogercresseyi* obtained from different geographical regions, using third-generation sequencing technology based on nanopore sequencing of full 16S rRNA gene. The results reported in this study are of high importance to support the development of sustainable salmon aquaculture worldwide, where the reduction of antibiotics and other drugs is pivotal in parallel with the characterization of potential reservoirs for fish pathogens.

## Results

### Nanopore sequencing output

**Figure 1:**
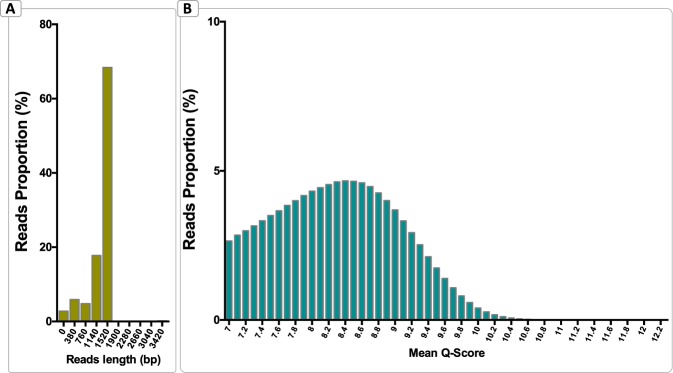
Distributions of reads according to sequence length (**A**), and distribution of reads according to their average Q-Score of quality (**B**).

A total of 4.945.864 reads passed the initial filter and were processed for taxonomical analysis. After the steps of demultiplexing, trimming, and length filter, an average of 94.6% were successfully classified in a taxonomical group (Supplementary Table S1) with at least 75% identity. All samples presented more than 300.000 reads except one sample from the Aysén region (ACS 30a) that presented fewer reads due to DNA quantity obtained from the sample. Since this study has a microbiome communities’ exploratory character, this sample was evaluated for the relative abundance of the identified taxa but was excluded from statistical analysis. The average sequence length was 1438 bp, but more than 60% of the sequence’s length was ranging from 1520 bp to 1900 bp (Fig. 1A), whereas the overall quality was 8.36 (Fig. 1B).

### *C*. *rogercresseyi* microbial community structure

**Figure 2:**
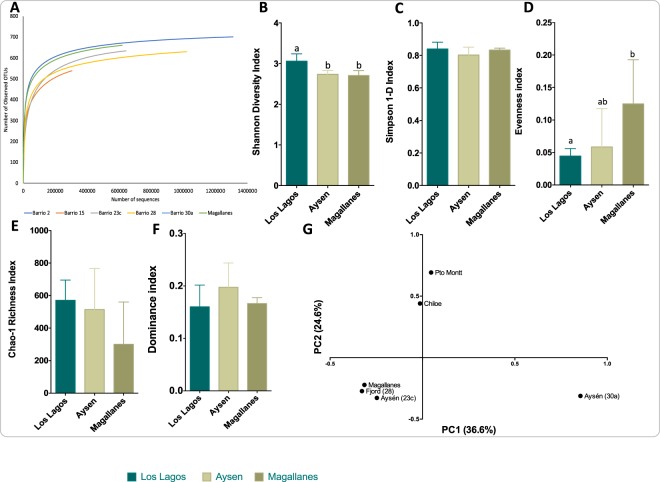
Diversity analysis of *Caligus rogercresseyi* microbiota community. Rarefaction curve (**A**), Shannon (**B**) and Simpson 1-D (**C**) diversity indices, evenness index (**D**), richness Chao1 estimator (**E**), dominance index (**F**) and principal coordinate analysis of the Bray Curtis distances between communities (**G**). Bars in B-F are mean±standard deviation and different letters indicate significant differences (One-Way ANOVA and post hoc Tukey HSD test, P < 0.05).

**Figure 3:**
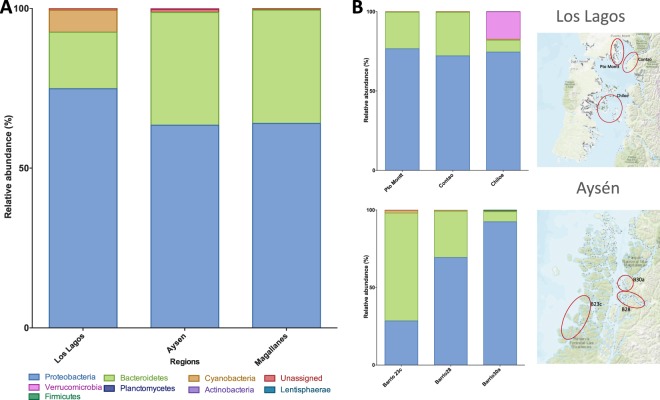
Relative abundance of *C*. *rogercresseyi* associated microbiota at phylum level by region (**A**), and among areas of Los Lagos (upper) and Aysén (lower) regions (**B**) (Map adapted from Daniel Jimenez).

**Figure 4:**
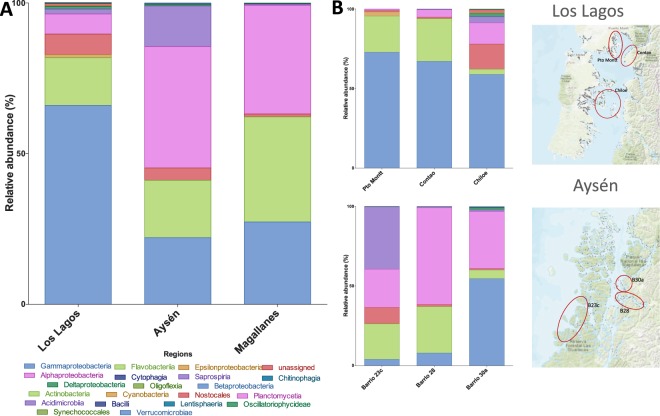
Relative abundance of *C*. *rogercresseyi* associated microbiota at class level by region (**A**), and among areas of Los Lagos (upper) and Aysén (lower) regions (**B**) (Map adapted from Daniel Jimenez).

**Figure 5:**
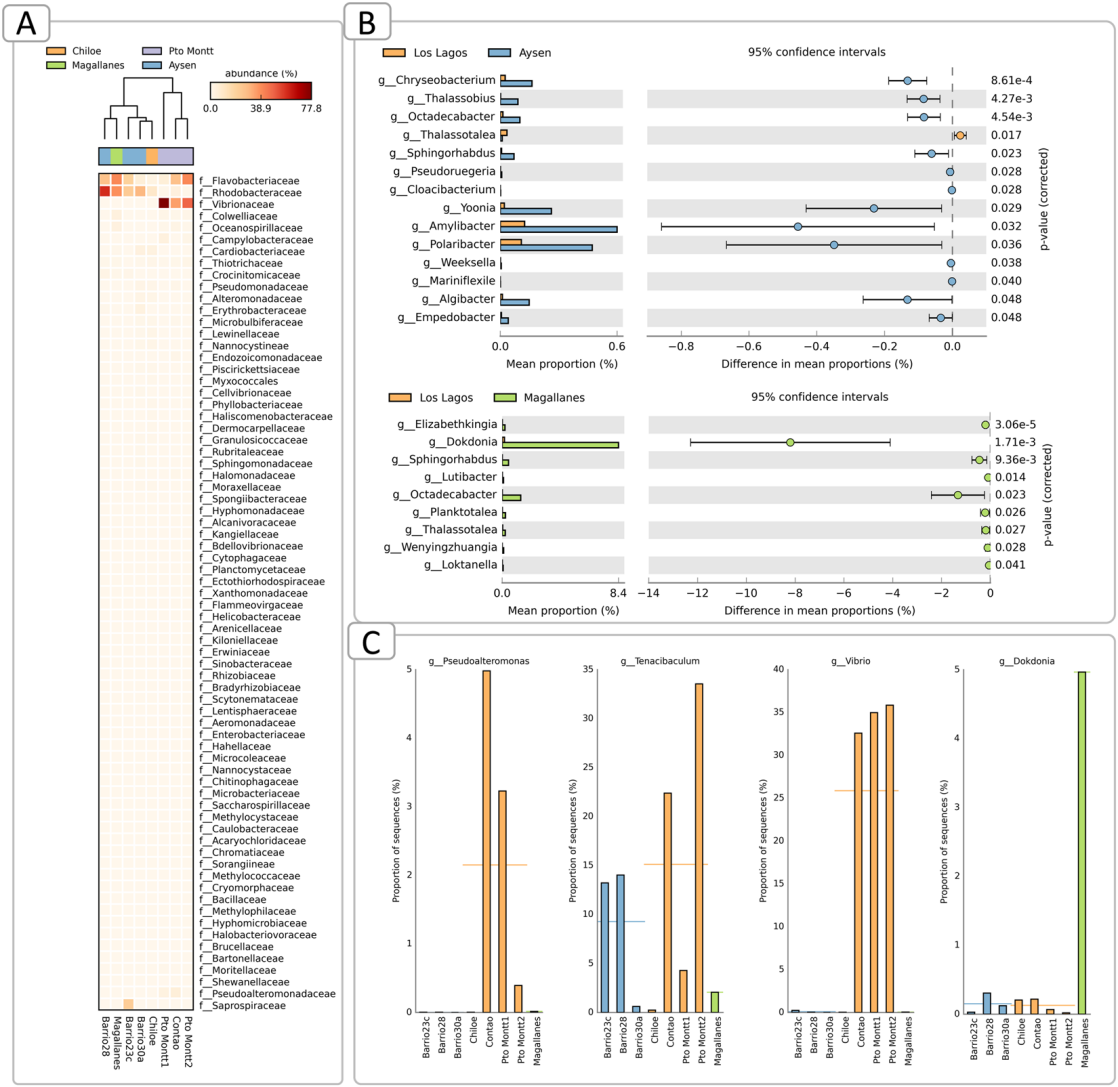
*Caligus rogercresseyi* associated microbial community characterization by hierarchical clustering at family level by UPGMA method (**A**); Comparison of microbiota relative abundance at genus level from Los Lagos, Aysén and Magallanes region and confidence intervals for the significative differences (**B**); Relative abundance of four known potential pathogens found with significant abundance in at least one region of the study (**C**).

Rarefaction analysis indicated that except for samples from the ACS 30a and ACS 15, samples reached a plateau denoting that the number of sequences was enough to cover all the identified taxonomical features (Fig. 2A). The microbial community associated with *C*. *rogercresseyi* from the Los Lagos region presented higher diversity than the communities from other regions, but this difference was significant only when measured by the Shannon diversity index (Fig. 2B) and not by Simpson’s 1-D index (Fig. 2C). Microbial community from the ectoparasite original from Magallanes presented higher evenness than the community from Los Lagos (Fig. 2D), as well as lower richness (Fig. 2E), although the latter was not significant. Based on the Bray-Curtis distances between bacterial communities, Los Lagos (Pto Montt and Chiloe) separated from others (Fig. 2G). The most abundant phyla were Proteobacteria with more than 60% prevalence regardless of region (Fig. 3A), followed by Bacteroidetes and Cyanobacteria. When comparing the three areas of Los Lagos, the microbial community of *C*. *rogercresseyi* from Chiloe presents a higher abundance of Verrucomicrobia and fewer Bacteroidetes, whereas more Bacteroidetes were found in the ACS 23’s community in Aysén (Fig. 3B). At class level, bacterial community from Los Lagos sea lice has high abundance of Gammaproteobacteria followed by Flavobacteriia and Alphaproteobacteria, whereas in Aysén and Magallanes the communities have less Gammaproteobacteria and more Alphaproteobacteria and Saprospira (in the case of Aysén) (Fig. 4A). Within Aysén, communities from different areas have different class abundances, with a higher abundance of Saprospira in ACS 23c, as well as a higher abundance of Alphaproteobacteria in ACS 28 (Fig. 4B). At family level, microbial communities of sea lice from Pto Montt and Contao (ACS 2, Los Lagos region) are more similar, whereas communities from Chiloe clustered with communities from Aysén and Magallanes (Fig. 5A). Microbiota from Magallanes sea lice clustered with the community obtained from sea lice from fish produced in the fjords at ACS 28. When comparing the communities by regions at the genus level, 14 genera were significantly different between Los Lagos and Aysén, and 9 between Los Lagos and Magallanes (Fig. 5B). However, when considering the different areas within each region other genus are significantly different (Fig. 5C) and these were *Pseudoalteromonas*, *Tenacibaculum*, and *Vibrio* with a significantly higher abundance in sea lice from Pto Montt and Contao, and also *Dokdonia* genus with higher abundance in Magallanes’ sea lice.

**Figure 6:**
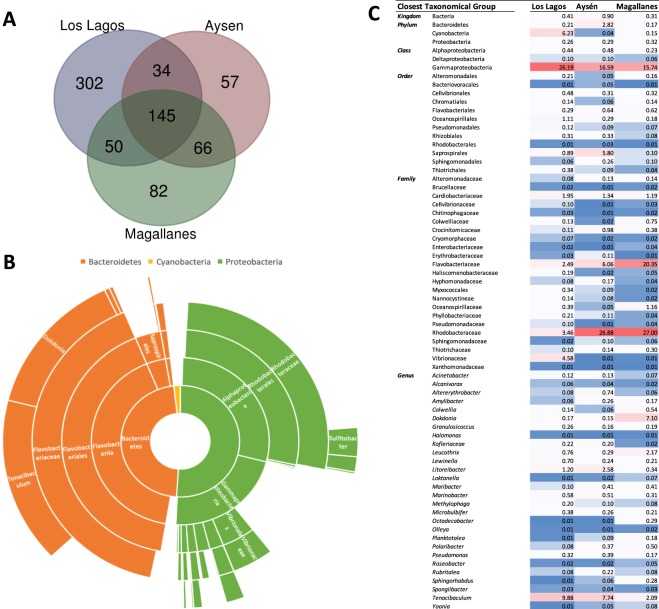
Caligus rogercresseyi core microbiota. Venn diagram representing the taxonomical features identified in the three regions of the study (**A**); Taxonomical distribution of taxa included in the 145 taxon shared by sea lice microbial community of the three regions based on the average relative abundance (**B**); Heat map of relative abundance of core microbiota taxa identified to the deepest level with a 95% confidence on identity, with colors ranging from blue to red representing lower to higher relative abundance respectively (**C**).

Microbial taxa present in sea lice form all regions was considered part of the core microbial community and included 145 taxa (Fig. 6A) that constitute 19.7% of the total identified taxonomical features. This community includes only three phyla: Proteobacteria, Bacteroidetes and Cyanobacteria. The first two were found in similar abundances, being the family Flavobacteriaceae predominant within Bacteroidetes, whereas within Proteobacteria elements of Rhodobacteraceae also Vibrionaceae were the most abundant (Fig. 6B). The abundance of the taxonomical features that comply with the *C*. *rogercresseyi* core community account for 70.3% of the obtained sequences from sea lice from the Los Lagos region, 86.8% of sequences from Aysén and 91.4% of sequences from Magallanes. Still, some features present different abundances between regions, and the most notable is the class Gammaproteobacteria which have higher abundance in sea lice from Los Lagos, Flavobacteriaceae and Rhodobacteraceae families with higher abundance in sea lice from Magallanes and the later also from Aysén, and the genus *Dokdonia* and *Tenacibaculum* with higher abundance in sea lice form Magallanes and from Los Lagos and Aysén, respectively (Fig. 6C). Moreover, the output of the mock community sequencing (Supplementary Table S2) used as internal technical control, indicated a good correspondence between the expected and the observed relative abundances.

## Discussion

The sea louse *Caligus rogercresseyi* is an ectoparasite that affects salmon farming in southern Chile, not only by impacting fish health and performance with consequent increase in productions costs, but also by contributing to environmental jeopardy through the massive application of drugs to control the infestation^6,33^. Knowledge of parasite’s biological systems and strategies is crucial to consolidate adequate responses for sustainable management of the disease. In terms of microbiota evaluation, high-throughput sequencing is currently used to reveal information that is hardly available by culture-dependent methods. In this study, we sequenced the full-16S rRNA gene using third-generation sequencing technology to characterize for the first time the microbiota associated with *C*. *rogercresseyi* infecting Atlantic salmon from different regions in Chile. This information is especially relevant in parasites since it has been conjectured that these communities might play a fundamental role in the copepod fitness^16^ and, therefore, in its capacity to resist host defenses and successfully infect.

Regarding diversity, Shannon’s index was higher in the community of microorganisms from sea lice obtained in the Los Lagos Region, and this might be related to the higher taxonomical features found only in sea lice from this region (>300, Supplementary Fig. 3S). On the other hand, although not significantly, in Magallanes, sea lice microbiota community is the least rich. From the three regions in the study, Los Lagos is most influenced by anthropogenic activities since it has several cities in the surroundings, and it was one of the first places where the commercial salmon aquaculture started with higher presence nowadays^33^. This fact contrasts with Magallanes, the most remote with less environmental pressure either anthropogenic or by aquaculture^33^. Notably, the first official *C*. *rogercresseyi* infestation report was recently archived^34^, suggesting some correlation between aquaculture pressure and *C*. *rogercresseyi* microbial community composition. However, more studies are required to explore the impacts of the sea lice microbiota in salmon farming and with particular emphasis to elucidate the capacity of lice’s microbiota to trigger infectious disease outbreaks.

More than 140 taxonomical features were identified in *C*. *rogercresseyi* core microbiota, and although these represent only 19% of the total features identified in the study, more than 70% of the sequences belonged to these core features, indicating the presence of robust resident microbiota in the parasite, which might be species-specific. Dalvin, *et al*.^18^ identified in *Lepeophtheirus salmonis* a dominance of members of the orders Flavobacteriales, Burkholderiales, Mycoplasmatales and Spirochaetales, and class Alphaproteobacteria in the associated microbiota. In *C*. *rogercresseyi* a high presence of Alphaproteobacteria and Flavobacteriales was also observed, but the other taxa, although present, were not highly abundant. On the other hand, contrary to what was described for *L*. *salmonis*^18^, in *C*. *rogercresseyi* microorganisms from orders Vibrionales and Alteromonadales were found in practically all samples. In another study conducted in *L*. *salmonis*, microorganisms from genus *Arcobacter*, *NS10* marine group, *Pseudomonas*, *Aeromonas*, *Vibrio*, *Rhizobiales*, and *Tenacibaculum* were identified^19^. Except for the first two above mentioned taxa, all the others were identified in *C*. *rogercresseyi*, which might indicate some level of similarities between microbiota associated to salmon ectoparasites.

The strong presence of Proteobacteria and Bacteroidetes in the core microbiota indicates dominance of Gram-negative bacteria, among which several known fish pathogens. It has been reported that the sea lice external surface area is covered by microorganisms^35^, and this includes pathogens from *Tenacibaculum*, *Pseudomonas* and *Vibrio* genus^36^, all of these found in *C*. *rogercresseyi* with noteworthy abundances. The origin of parasite’s microbiota is expected to be the host mucus and skin (the parasite’s dietary sources) and milieu water^19^. However, host mucus microbiota composition is altered by many factors such as salinity^37^, diet^38^, and even the simple presence of the parasite^19^. Therefore, it would be expected that these modulations would impact sea lice microbiota composition as well. However, reports indicate that sea lice and salmon mucus microbiota are not necessarily correlated^19^, and this supports the findings of this study where a stable, robust core microbiome was identified regardless region, center, producer practices or other factor. The factors influencing the stability of the core community are yet to be unraveled, but it is known that other parasites such as the nematode *Heligmosomoides polygyrus* can secrete antimicrobial compounds that shape the host-microbiota for the parasite’s benefit, increasing its fitness^39^. It is known that sea lice developed a complex secretome^40^, that modulates host immune response^41^, and host mucus microbiome^19^.

The functional role of the identified core microbiota is of high interest for future studies since its potential to enhance sea lice fitness, and resistance is high. For instance, microbiota in insects actively participates in the peritrophic matrix – a chitin and proteins layer that surrounds gut epithelium and is present also in crustacean species – and this is thought to protect the host by reducing the influx of undesirable bacteria to the body^42^, possibly preventing an exacerbated immune response from host (in this case sea lice) to pathogenic bacteria present in the environment. On the other hand, adding to the high prevalence observed in pathogenic bacteria, *C*. *rogercresseyi* microbiota also showed a strong presence of taxa known as bioactive compounds producers. For example, organisms from genus *Cobetia* are nonpathogenic but produce biofilm and secrete exopolysaccharides, as well as microorganisms from the *Dokdonia* genus, a highly prevalent genus in Magallanes sea lice microbial community. Biofilm production is a characteristic that confers adhesion, protection, and robustness to the bacteria^43^, and might be influencing the stability of the microbiota composition and fitness of the community. Members from the genus *Pseudoalteromonas* are microorganisms with the capacity to secrete bioactive compounds with antialgae and antimicrobial properties^44^, and these were found in higher abundance in sea lice from Pto Montt and Contao in Los Lagos region. These are examples of the complex set of bioactive compounds producing microbiota present in *C*. *rogercresseyi* that might be increasing the parasite’s resistance and persistence.

Using third-generation sequencing technology by Nanopore MinION in aquaculture is a novel approach that produced a meaningful set of information on a relevant pathogen for the sector. Despite the increasing amount of studies being reported using the technology released in 2014, this is the first study in an aquatic invertebrate. The higher error rate of nanopore sequencing compared to other platforms (i.e. Illumina) has been one of the main issues limiting the dissemination of this technology to broader fields. In microbial analysis however, several studies have revealed that the error rate has been somehow compensated by reads length, generating outputs comparable to Illumina^26,27,45–48^, sharing more than 95% of total taxonomical abundance^49^. These studies support the robustness of the results achieved with this technology and together with the low initial cost and high portability of the device, suggests that it will be a preferred to other technologies for microbial studies. Sequencing the full 16S rRNA gene increases identification power^47^, and indeed in this study we achieved a high level of species identification indicating its potential for aquaculture pathogen detection or future monitoring and control of microbial dynamics.

## Conclusions

Overall, the microbial composition of *C*. *rogercresseyi* is dominated by gram-negative microorganisms, including several pathogens and taxa associated with biofilm and bioactive compounds production, and these characteristics might be related to the high prevalence of the parasite and its resilience capacity. The presence of a stable and highly abundant core microbial community suggests a link to parasite fitness, and further research should be a pursuit to understand the role of these microorganisms in the maintenance of the core microbiota and its relationship with the parasite success and life strategies. Several taxa with bioactive compound secretory capacity were identified, suggesting a possible role during the host infestation processes. The microbiota community was differentially associated with the salmon production, where several potential pathogens in Los Lagos, Aysén and Magallanes region were identified. The putative role of sea lice as vectors for fish pathogens and also reservoirs for antibiotic-resistant genes should be further investigated.

## Methods

### Preliminary evaluation

The first step of this evaluation was to perform DNA extraction. Although different extraction kits and methods have been compared in other studies, the presence of exoskeleton in organisms such as copepods represents a challenge not usually addressed by the existent protocols^50^. Most of the studies focused on the evaluation of symbiont microbiota in animals use fecal contents as biologic material, and in organisms such *as C*. *rogercresseyi*, such material is hardly accessible. Microbiota associated with gut tissue has been evaluated in insects by dissecting the abdomen segments that held the full digestive tract^51,52^. This type of dissection requires equipment and technical precision that is not available when sampling in the field where conditions are not stable (as in the example in a salmon farm sea cages), and in these cases, its preferable using the whole animal to assure procedures homogeneity. According to Rubin, *et al*.^50^, the DNA extraction method used in terrestrial arthropods did not affect the identification of the bacterial community structure of the organisms but affected the capacity of the sample to be successfully sequenced. This enhances the importance of optimizing extraction protocols before sequencing. Therefore, previously to sequencing, several preliminary tests were performed to select the DNA extraction method, and the minimum number of copepods to be pooled in a sample. To evaluate different DNA extraction methods, adult females of *C*. *rogercresseyi* from the wet lab of the National Reference Laboratory for this ectoparasite were obtained. Five protocols were tested; (a) commercial kit Qiagen DNeasy Fast Mini Stool (Qiagen, USA) with slight modification increasing incubation time and temperature, (b) commercial kit Qiagen DNeasy Blood and Tissue (Qiagen, USA) with modifications according to Michl, *et al*.^53^ that includes a lysis buffer with lysozyme to increase Gram-positive bacteria DNA yield, (c) commercial kit Qiagen DNeasy Blood and Tissue with the manufacturer’s modification for insects, (d) salt-extraction^54^ and (e) salt-extraction protocol modified to include column purification and more extended incubation period. During a previous study, we identified difficulties in the complete dissociation of the copepod’s exoskeleton using only mechanical disruption with ceramic beads, as previously mentioned by Rubin, *et al*.^50^, and these were surpassed by increasing incubation periods. Regardless of the extraction method used, pools of 10 females preserved in 99–100% ethanol (molecular grade) were washed in sterilized by autoclave and UV PBS (phosphate-buffered saline solution) and were cut in small pieces in an ice cold sterilized petri dish. The lysis method was dependent on the protocol under analysis, and the five methods included a negative control with no sample and positive control with 10 mg of a commercial mixture of two *Bacillus subtilis* strains. Extracted DNA was measured by spectrophotometry in a Nanodrop (ThermoScientific, USA), and integrity was verified by a TAE (Tris-Acetic Acid-EDTA buffer) gel electrophoresis with 1% agarose. The presence of bacterial DNA after each extraction was evaluated by amplification of the V4 hypervariable region of the 16S rRNA gene using the universal primers 515F- GTGCCAGCMGCCGCGGTAA, 806R-GGACTACHVGGGTWTCTAAT in a PCR with 50 ng of extracted genomic DNA as template in a Verity thermal cycler (Applied Biosystems, USA), producing a 390 bp amplicon as described in Gonçalves and Gallardo-Escárate^55^. Extracted DNA purity and the qualitative evaluation of the intensity of PCR amplicon was used to decide the most appropriate method, and overall results are presented in Supplementary Fig. 1S. After choosing DNA extraction protocol, extraction was performed in triplicate to a single *C*. *rogercresseyi* adult and pools of two, three, and four specimens to obtain genomic DNA that was after used as a template in the above-described PCR for V4 amplification.

### *Caligus rogercresseyi* sampling and sample processing

**Figure 7:**
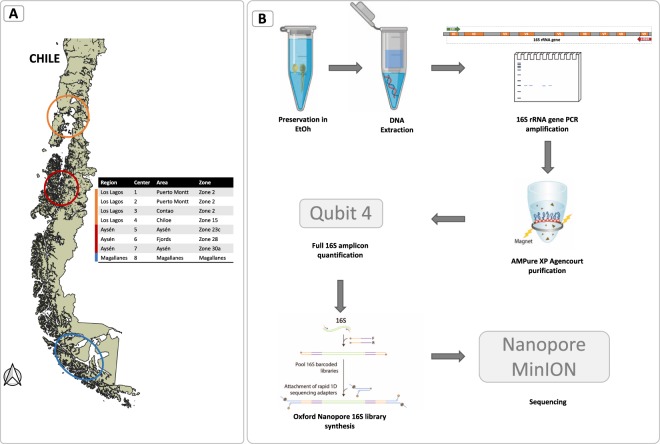
Chile’s partial map with identification of sampling regions (**A**), and experiment workflow diagram (**B**) (Map adapted from Daniel Jimenez).

Parasites were obtained from commercial salmon farms from the three regions with active industrial productions of salmon in Chile, Los Lagos (or X Region), Aysén (or XI Region) and Magallanes (Fig. 7A and Supplementary Fig. 2S). Samples were collected during Spring and Summer and centers were selected based on a) geographical distribution to include centers that would represent distinct aquaculture areas (denominated as “ACS” or Salmon farming concessions grouping by Chilean authorities) of the three major regions, b) production cycle in order to avoid centers with less than 8 months of seawater production that could still be under the influence of any antilousing prophylaxis administered during freshwater stage (e.g. Lufenuron), and c) drugs utilization, preferring centers with less or no drug (both delousing and antibacterial) usage in the cycle. Following this, sampling was performed in four centers of the Los Lagos region, three centers in the Aysén region, and one center in the Magallanes region. Samplings were performed during the morning, and procedures were previously standardized to ensure reproducibility between centers. When possible, samples were collected during the sampling performed by the centers as part of the compulsory sea lice counting required by Chilean authorities (Sernapesca), and procedures were performed according to sampling protocols approved by the same entity. Atlantic salmons (*Salmo salar*) were confined in the sea cages using secondary nets and were collected with a soft net and sedated in a reservoir previously loaded with Benzocaine (20%) in seawater (15 ml per 100 L seawater). After approximately 2 minutes, fish were examined for parasites, and these were collected with plastic sterilized forceps and placed in vials with molecular grade ethanol. Vials were stored at −20 °C and transported in cooled boxes by overnight express service to our laboratory where they were stored at −80 °C until processing.

All operations involving fish handling were approved by the Ethical Committee of the University of Concepción and by the Chilean Authority SERNAPESCA, that regulates all operations in culture centers. *Caligus rogercresseyi* is a copepod, and since regulations for animal use in research do not cover this taxon, no ethical approval of these experiments was required. All methods and procedures were performed in accordance with the relevant guidelines and regulations.

### DNA extraction

Pools of 10 adults were thawed, washed and sliced as above detailed and were processed as described in the study workflow (Fig. 7B), starting with DNA extraction following the protocol that presented the best results during the preliminary study. The minced copepods were mixed with 1 mL of lysis buffer (10 mM Tris-HCl, 400 mM NaCl, 100 mM EDTA, 0.4% SDS, and 24 ug/mL Protease K, pH 8.0), after which were thoroughly vortexed and incubated overnight and 56 °C. The contents of each tube were centrifuged at 5000 rpm for 1 min and supernatant mixed with molecular grade 99–100% ethanol and then added to a silica column that was centrifuged at 8.500 rpm for 1 min. The column was washed with the kit buffers (including a buffer with a low concentration of a guanidine based chaotropic salt), and after complete centrifugation, DNA was eluted in two steps of 15 uL of DEPC water finalizing with a total volume of 30 uL eluted DNA. DNA integrity was verified by electrophoresis as above mentioned, and concentration measured by fluorescence in a Qubit 4 (ThermoScientific, USA) using the Qubit dsDNA BR Assay Kit (ThermoScientific, USA) following the manufacturer’s instructions. Genomic DNA samples were diluted to 100 ng/uL and amplification of full-16S rRNA gene was performed by PCR with reaction volume of 25 uL, using the primers 27 F 5′-AGAGTTTGATCCTGGCTCAG-3′ y 1492 R 5′ GGTTACCTTGTTACGACTT-3′, using Taq DNA polymerase LongAmp (NewEngland Biolabs, USA), with an initial denaturing step at 95 C for 1 min, followed by 25 cycles of 95 °C for 20 sec, 56 °C for 30 sec and 65 °C for 2 min, and an extension at 65 °C for 5 min. PCR products were evaluated by electrophoresis, as previously described.

### Nanopore library synthesis and sequencing

After confirming the presence of full-16S rRNA amplicon, PCR products were purified with Agencourt AMPure XP beads (Beckman Coulter, USA) in a 1:2 sample-beads ratio. The mixture was incubated for 5 min at room temperature, and the sample placed in the magnetic stand for ethanol washing (freshly prepared at 70%). The washed mixture was resuspended in ultrapure sterilized water, and the purified product was separated from beads by placing it in a magnetic stand. The purified amplicon obtained from pooled *C*. *rogercresseyi* DNA was quantified again in the fluorometer Qubit 4 (ThermoScientific, USA) and was used as a template for library synthesis using the 16S Barcoding Kit (SQK-RAB204, Oxford Nanopore Technologies), following the manufacturer’s instructions for 1D sequencing strategy. Purified 16S amplicon was mixed with barcodes accordingly, and using the LongAmp Taq Polymerase (New England Biolabs), a PCR was performed as described in the previous step, with a final reaction volume of 50 uL. The PCR product was purified again with Agencourt AMPure XP beads and incubated in HulaMixer for 5 min at room temperature. After the magnetic beads washing step of the purified product was eluted in 10 uL of elution buffer (10 mM Tris-HCl pH8.0 with 50 mM NaCl). The final concentration of the library was quantified in fluorometer Qubit 4 (Thermoscientific, USA), and libraries were evaluated on a TapeStation Bioanalyzer 2100 (Agilent, USA), using DNA ScreenTape (Agilent, USA) following the manufacturer’s instructions. A single library was synthetized from DNA of a pool of ectoparasites per center, and libraries were pooled in multiplex mode following the protocol of Oxford Nanopore Technologies, and the flowcell MK1 Spot-ON FLO-MIN107-R9 was loaded accordingly. Sequencing efficiency was monitored through the software MinKNOW 2.0 (Oxford Nanopore Technologies). Finally, as internal control, the DNA of a microbial mock community (ZymoBiomics Microbial Community Standard) was extracted and sequenced following the same describe procedures and observed abundances of taxa were compared with expected abundance.

### Bioinformatic analysis

After the sequencing run, generated fast5 files were base-called using Guppy (version3.2.2, Oxford Nanopore Technologies, UK), and a filter step was applied to retain only sequences with Q-score ≥7 (quality filter). Sequences were then evaluated using the Fastq 16S v3.2.1 (Oxford Nanopore Technologies, UK) pipeline that included demultiplexing and primers and barcode trimming. Sequences with ambiguous identification were excluded from subsequent analysis. BLASTN aligned cleaned sequences against NCBI RefSeq for 16S ribosomal RNA database for bacteria and archaea within the EPI2ME software package (Oxford Nanopore Technologies), and only the sequences with identity ≥75% were retained (confidence level filter) as mentioned by Edwards, *et al*.^23^. The occurrence of each NCBI taxonomy identifier was evaluated for each sample using R^56^, and the relative abundance of each NCBI taxa id was calculated per sample to normalize. Taxonomical identifiers were ordered, and to minimize singletons, doubletons product of sequencing errors taxon with <0.01% of relative abundance in at least one sample were eliminated. For each sample, an equivalent to OTU (Operative Taxonomic Unit) frequency table was built using R, and rarefaction analysis were performed to assess sequencing depth performance. The alpha diversity of microbiota communities associated with *C*. *rogercresseyi* was compared between region by calculating the Shannon and Simpson 1-D diversity indices, the Chao-1 richness index, community Evenness, and dominance index using PAST3 v3.25^57^. Differences between regions were evaluated with One-Way ANOVA followed by the posthoc multiple comparison Tukey-HSD test when applicable, considering a P-value < 0.05. Microbial community structure was analyzed using Bray-Curtis distances based on taxon relative abundance data, and principal coordinate analysis (PCoA) were performed as a multivariate unsupervised data exploration. Variations of the microbial community along geographical distribution were explored by plotting relative abundance at Phylum and Class level, and multivariate exploratory analysis was performed through Principal Component Analysis of the OTU’s relative abundance at the order level.

Distance between communities of different areas was evaluated at family level by hierarchical clustering using UPGMA method, and genus differential abundances between regions were evaluated by Welch’s t-test considering an interval of confidence based on the Newcombe-Wilson method^58^ with P-value correction of Storey’s FDR^59^. When applicable, comparisons of bacterial communities’ taxon abundance were performed between two samples (i.e., between two areas) by assessing the Fisher’s exact test^60^ with P-value correction of Storey’s FDR. Besides, to complement the first assessment of *C*. *rogercresseyi* associated microbiota communities, the “core” microbiome was defined as the set of OTU’s present in samples from all the geographical regions sampled in the study, and these OTU’s were evaluated following the deepest taxonomical assignation with an identity ≥95%.

## Supplementary information

Supplementary Information.

## Data Availability

Data are available at NCBI-SRA BioProject PRJNA598707.
