# Supplementary material for: Nanopore sequencing of microbial communities reveals the potential role of sea lice as a reservoir for fish pathogens
Source: Sci Rep. 2020 Feb 19;10:2895. doi: 10.1038/s41598-020-59747-0 (PMC7031262; doi:10.1038/s41598-020-59747-0)
Supplement: Supplementary file 1 — Supplementary Information. [file 41598_2020_59747_MOESM1_ESM.docx]

SUPPLEMENTARY INFORMATION

**Nanopore sequencing of microbial communities reveals the potential role of sea lice as a reservoir for fish pathogens**

Ana Teresa Gonçalves^1,2^, Rayen Collipal-Matamal^2^, Valentina Valenzuela-Muñoz^1,2^, Gustavo Nuñez-Acuña^1,2^, Diego Valenzuela-Miranda^1,2^, Cristian Gallardo-Escárate^1,2^*

^1^Interdisciplinary Center for Aquaculture Research, University of Concepción, Concepción, Chile.

^2^Laboratory of Biotechnology and Aquatic Genomics, Center of Biotechnology, University of Concepción, Concepción, Chile.

*corresponding author: Cristian Gallardo-Escárate (crisgallardo@udec.cl)

500 bp

V4 region of 16S rRNA

**Figure 1S A.** Amplification of the V4 region of 16S rRNA gene from DNA purified with different protocols (A-E). Cal represents *Caligus rogercresseyi*; Bac represents mixture of *Bacillus subtilis* strains and C(-) represents PCR negative control.


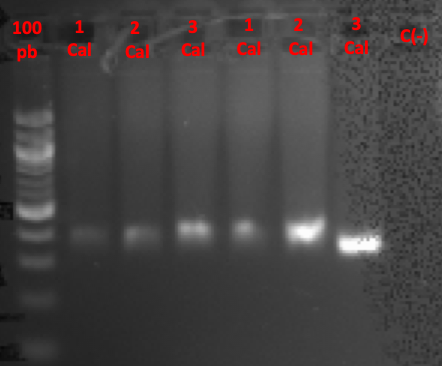


500 bp

V4 region of 16S rRNA

**Figure 1S B.** Amplification of V4 region of 16S rRNA gene with DNA extracted from 1, 2 or 3 adult females of *C. rogercresseyi* from different populations.

**Figure 2S**. Geographical detail of the sampling areas. A) Sampling areas from Los Lagos region: ACS 2 and ACS 15 with red circle, and B) sampling areas from Aysén region: ACS 23c, 30a and 28 marked with red circle (Map adapted from Daniel Jimenez).

**Figure 3S**. Venn diagram of exclusive and shared taxonomical unique features identified in *Caligus rogercresseyi* associated microbiota by area: Left venn represents areas from Los Lagos regions, right venn represents areas from Aysén region, and central diagram represent all features from the three regions of the study, identifying 145 features as the core microbiota community of the copepod.

| **Table S1**. Nanopore MinION full-16S rRNA sequence output for *Caligus rogercresseyi* from different Chilean regions | | | | | | |  |
| --- | --- | --- | --- | --- | --- | --- | --- |
| **Region** | **Center location** | **Area** | **Reads passed** | **Average length** | **Average Q-score** | **Reads with taxonomy (%)** | |
| Los Lagos | Puerto Montt | ACS 2 | 619.910 | 1476 | 8.56 | 95.8 |  |
|  | Puerto Montt | ACS 2 | 680.727 | 1643 | 8.96 | 95.6 |  |
|  | Contao | ACS 2 | 767.987 | 1847 | 8.54 | 93.2 |  |
|  | Chiloe | ACS 15 | 380.485 | 1543 | 8.65 | 95.1 |  |
| Aysén | Aysén | ACS 23c | 688.690 | 1423 | 8.43 | 93.5 |  |
|  | Fjords | ACS 28 | 1.109.414 | 1786 | 8.38 | 93.1 |  |
|  | Aysén | ACS 30a | 10.887 | 1656 | 8.98 | 94.6 |  |
| Magallanes | Magallanes | ACS 57 | 687.764 | 1549 | 8.65 | 95.7 |  |

*ACS = Salmon farming concessions grouping.

| **Table S2.** Observed abundance of Nanopore sequencing of bacterial mock community | | |
| --- | --- | --- |
| **Species** | **Expected abundance (%)^a^** | **Observed abundance (%)^b^** |
| *Pseudomonas aeruginosa* | 4.2 | 6.6 |
| *Escherichia coli* | 10.1 | 14.5 |
| *Salmonella enterica* | 10.4 | 13.1 |
| *Lactobacillus fermentum* | 18.4 | 14.0 |
| *Enterococcus faecalis* | 9.9 | 8.4 |
| *Staphylococcus aureus* | 15.5 | 12.7 |
| *Listeria monocytogenes* | 14.1 | 13.9 |
| *Bacillus subtilis* | 17.4 | 14.9 |
| Others (prokaryote) | na | 1.8 |
| ^a^Expected abundance by 16S sequencing according to the provider of mock community (ZymoBiomics Microbial Community Standard); ^b^Observed abundance after Nanopore sequencing and bioinformatic pipeline used in this study, and data are % of reads abundance in relation with total reads (1.062.870 reads). | | |
